# Assessment of gene order computing methods for Alzheimer's disease

**DOI:** 10.1186/1755-8794-6-S1-S8

**Published:** 2013-01-23

**Authors:** Benqiong Hu, Gang Jiang, Chaoyang Pang, Shipeng Wang, Qingzhong Liu, Zhongxue Chen, Charles R Vanderburg, Jack T Rogers, Youping Deng, Xudong Huang

**Affiliations:** 1 College of Management Science, Chengdu University of Technology, Chengdu 610059, China; 2 Group of Gene Computation, College of Mathematics and Software Science, Sichuan Normal University, Chengdu 610066, China; 3 Department of Computer Science, Sam Houston State University, Huntsville, TX 7734, USA; 4 Department of Epidemiology and Biostatistics, School of Public Health, Indiana University Bloomington, 1025 E. 7th Street, Bloomington, IN 47405-7109, USA; 5 Harvard NeuroDiscovery Center and Department of Neurology, Massachusetts General Hospital and Harvard Medical School, Charlestown, MA 02129, USA; 6 Neurochemistry Laboratory, Department of Psychiatry, Massachusetts General Hospital and Harvard Medical School, Charlestown, MA 02129, USA; 7 Cancer Bioinformatics, Rush University Cancer Center, and Department of Internal Medicine, Rush University Medical Center, Chicago, IL 60612, USA

## Abstract

**Background:**

Computational genomics of Alzheimer disease (AD), the most common form of senile dementia, is a nascent field in AD research. The field includes AD gene clustering by computing gene order which generates higher quality gene clustering patterns than most other clustering methods. However, there are few available gene order computing methods such as Genetic Algorithm (GA) and Ant Colony Optimization (ACO). Further, their performance in gene order computation using AD microarray data is not known. We thus set forth to evaluate the performances of current gene order computing methods with different distance formulas, and to identify additional features associated with gene order computation.

**Methods:**

Using different distance formulas- Pearson distance and Euclidean distance, the squared Euclidean distance, and other conditions, gene orders were calculated by ACO and GA (including standard GA and improved GA) methods, respectively. The qualities of the gene orders were compared, and new features from the calculated gene orders were identified.

**Results:**

Compared to the GA methods tested in this study, ACO fits the AD microarray data the best when calculating gene order. In addition, the following features were revealed: different distance formulas generated a different quality of gene order, and the commonly used Pearson distance was not the best distance formula when used with both GA and ACO methods for AD microarray data.

**Conclusion:**

Compared with Pearson distance and Euclidean distance, the squared Euclidean distance generated the best quality gene order computed by GA and ACO methods.

## Background

### A brief introduction of Alzheimer's disease

Being the most common form of age-related dementia, Alzheimer's disease (AD) affects 5.4 million Americans, and at least $183 billion will be spent in 2011 on care of AD and other dementia patients. The problem is worsening as life expectancy continues to increase. By 2050, the projected number of AD patients could range from 11 to 16 million people in the United States alone if no cure or preventive measure for AD is found. Hence, AD has quickly become a pandemic and exacted a huge socioeconomic toll [1].

AD is named after Dr Alois Alzheimer, who has first investigated the disease [2]. Later on, the autopsies of brain examinations of most cases of senility under light microscope were discovered to be extracellular deposits of β-amyloid and intracellular deposits of neurofibrillary tangles (NFTs). Abundant amounts of these lesions in the brain were necessary for a confirmed diagnosis of AD [3]. In 1984, an possible AD-related gene on chromosome 21 was implied when Glenner and Wong reported on the amino acid sequence of the main component of β-amyloid-, an approximate 4.3 kD peptide that they coined as "amyloid-β protein"(Aβ) based on their analysis of cerebrovascular amyloid derived from patients with Down's syndrome [4]. This study has laid the foundation for AD's "amyloid hypothesis" which claims that the accumulation of Aβ, as determined by its generation versus clearance in the brain, is the primary driver of AD-related pathogenesis, including neuronal cell death.

Frangione et al reported on the sequencing of the exons 16 and 17 of amyloid precursor protein (APP) to reveal the first pathogenic mutation in APP [5]. Finally the subsequent sequencing of these same two APP exons (encoding the Aβ portion of the molecule) that were truly linked to chromosome 21 led to the discovery of the first AD-related mutation [6]. Following this finding, Pericak-Vance and colleagues reported a significant genetic linkage of the more common late-onset of AD (> 65 years) to chromosome 19 [7]. Then in 1993, they found a common polymorphism in the gene encoding Apolipoprotein E (APOE)- APOE allele 4, is associated with increased risk for AD [8]. In 1993, the first study aimed at investigating the Presenilins as putative AD genes offered evidence for a significant association between a single-nucleotide polymorphism (SNP) in intron 8 of the Presenilin 1 (PSEN1) gene and AD. Estimates were that the common variants in PSEN1 could account for nearly half of the population-attributable risk for AD than was found for the APOE4 allele [9]. Then in 2001, a report investigating a consecutive series of referral-based AD cases found coding sequence mutations in 11% of the samples, suggesting that PSEN1 mutations may indeed be more frequent in the general population than had been previously assumed [10,11]. Furthermore, reports indicated that changes in the promoter region could lead to an altered expression pattern of the protein in neurons [12].

Currently, the mainly proposed therapeutic intervention for AD is anti-amyloid approach, which ranges from interdicting amyloidogenic processing of the β-amyloid precursor protein (APP) to removing amyloid plaques in the brain [13]. In addition to therapies based on curbing the production of Aβ or enhancing its clearance, another therapeutic strategy would be aimed at attenuating Aβ toxicity and neuroinflammation in the AD brain. Perhaps, the most effective way to approach the blocking of Aβ toxicity would be to prevent the formation of neurotoxic Aβ oligomers [3,14]. As APP, the Presenilins, and APOE represent the only firmly established AD genes to date for AD, they represent the most effective means of curbing the production of Aβ or accelerating the clearance and degradation of this peptide in the brain [3]. The identification of the remaining genes involved in AD will enable investigators and clinicians to further delineate the path of biological events that lead to AD-related neurodegeneration [3].

### Introduction of gene clustering and gene order

Having been applied to many biological domains, such as drug discovery, molecular diagnosis, and toxicological research, DNA microarray technology is used most importantly to generate gene data, which holds a lot of biological information. One common data structure of a microarray data set is the presentation of a matrix. In matrix *X*, element *X_ij _*represents the expression level of the *i-th *gene in the *j-th *experiment. Then the *i-th *line vector of matrix *X *represents a group of expression levels of the *i-th *gene. The *i-th *line vector contains the biological information of the *i-th *gene, and it is often used as an atom object of data to be processed.

One important aspect of biology is to make similar genes cluster together. Since line vectors of a matrix contain the information of genes, clustering similar vectors together is equivalent to cluster similar genes together. A number of algorithms were proposed to cluster gene expression profiles. Eisen *et al*. [15] applied hierarchical clustering [16], a widely used tool [17-20], to solve the problem. It also has some variants [21,22]. Self-organizing maps (SOMs) [23,24] and k-means clustering [25] were also used for the same purpose. Ben-Dor *et al*. [26] developed an algorithm- cluster affinity search technique (CAST), that has a good theoretical basis. Merz and Zell [27] proposed a memetic algorithm for the problem, formulated as finding the minimum sum-of-squares clustering [28,29].

To achieve a much better quality of clustering, the computing concept of gene order has been proposed. Gene order is the permutation of all line vectors in such a way that all the line vectors are ordered one by one in a sequence, and that similar vectors are ordered together. A gene is associated with a line vector of a matrix. The optimal gene order refers to the permutation that results in a sequence that all the vectors line up via the minimal distance. Alternatively, computing optimal gene order is equivalent to identifying a route of the traveling salesman problem (TSP) in which every vector associates with a gene that has been abstracted as a virtual city [30-35].

Since TSP is an NP-hard problem, the computation of the optimal gene order is NP-hard and only the approximation of the optimal gene order can be calculated. To obtain the approximation of the optimal gene order, Tsai *et al*. applied a family competition genetic algorithm (FCGA) [33-36] and Seung-Kyu *et al*. applied a hybrid genetic algorithm (NNGA) [37].

### Introduction of ant colony optimization (ACO)

First introduced in 1992, ant colony optimization (ACO) is a novel nature-inspired method based on the foraging behavior of real ants to solve TSP. (Dorigo, 1992; Dorigo *et al*., 1996, 1999; Dorigo and Stützle, 2004) [38]. When searching for food, ants initially explore the area surrounding their nest in a random manner. As soon as an ant finds a food source, it evaluates it and carries some food back to the nest. During the return trip, the ant deposits a pheromone trail on the ground. The pheromone deposited, the amount of which may depend on the quantity and quality of the food, guides other ants to the food source. As it has been shown (Goss *et al*., 1989), indirect communication among ants via pheromone trails enables them to find the shortest paths between their nests and food sources. ACO generates the TSP route of the highest quality in general compared with other methods. However, it is a challenge to apply ACO to calculating gene order; its running time has been too long even for input data that has less than 1000 elements when a common personal computer is used. To make ACO better suited for the computation of gene order, we have improved its running speed by factors of at least 200 [39,40].

### Introduction of genetic algorithm

Genetic algorithm (GA) can be understood as an intelligent probabilistic search algorithm that works on Darwin's principle of natural selection and that can be applied to a variety of combinatorial optimization problems [41]. More to the point, GAs are based on the evolutionary process of biological organisms in nature about which theoretical foundations were originally developed by Holland [32]. During the course of evolution, natural populations evolve according to the principle of natural selection and "survival of the fittest". Individuals who are more successful in adapting to their environments will have a better chance of surviving and reproducing, whilst individuals who are less fit will be eliminated.

To understand the outline of GA as in [42], the following original statement is given:

A GA simulates these processes by taking an initial population of individuals and applying a genetic algorithm to their reproduction. In optimization terms, each individual in the population is encoded into a string or chromosome that represents a possible solution to a given problem. The fitness of an individual is evaluated with respect to a given objective function. Highly fit individuals or solutions have opportunities to reproduce by exchanging pieces of their genetic information, in a crossover procedure, with other highly fit individuals. This produces new "offspring" solutions (i.e., children), who share some characteristics taken from both parents [43].

To date, there are few types of tools to calculate gene order. In our knowledge, GA [35] and ACO [39] are mostly used methods. Our study intends to address this question- which method is a better for AD gene order computation using AD microarray data under different conditions. Herein, we reported that ACO fits the AD microarray data the best when calculating gene order in comparison to the GA methods tested in this study.

## Methods

This study intends to answer the question of which algorithm, between ACO and GA, generates the optimal AD gene order. The distance formula, which measures the similarity degree of two genes, is the key parameter that affects the quality of gene order. With different distance formulas (see the following Formulae 1-3), the gene orders will be calculated using the tools of ACO and GA in this section. Then, the quality of gene order will be measured both by the fitness function and by a heat map.

### Traveling salesman problem (TSP)

TSP is introduced below:

Assume that there are *n *cities and a distance matrix *D *= [*d_ij_*], where *d_ij _*is the distance between city *i *and city *j*, and TSP is the problem of finding a permutation *π *of all the cities such that minimizes $\sum_{i=1}^{n-1}d_{\pi \left(i\right),\pi \left(i+1\right)}+d_{\pi \left(n\right),\pi \left(1\right)}$.

### Measurement of gene similarity

As aforementioned, a gene associates with a vector and the similarity of two genes can be estimated by the distance between the two vectors.

For two genes, different metric measurements will measure out different degrees of possible similarity. That is, the estimation of gene similarity is sensitive to the distance formula.

Many distance formulas of vectors to measure the similarity of genes are presented, such as Pearson correlation, absolute correlation, Spearman rank correlation [44], Kendall rank correlation [45], and Euclidean distance. In this paper, three popular distance formulas are introduced below.

The first distance measure is the Pearson correlation:

Let k-dimensional vector *X *= (*x*_1_, *x*_2_, ..., *x*_*k*_) and *Y *= (*y*_1_, *y*_2_, ..., *y*_*k*_) be the expression levels of two genes *X *and *Y*, which are observed over a series of *k *conditions. The Pearson correlation of two genes *X *and *Y *is

$$s_{X,Y}=\frac{1}{k}\sum_{i=1}^{k}\left(\frac{x_{i}-\bar{X}}{\sigma_{X}}\right)\left(\frac{y_{i}-\bar{Y}}{\sigma_{Y}}\right)$$

, where $\bar{X}$ and *σ_X _*is the mean and the standard deviation of the expression levels, respectively. The value of *σ_X _*is

$$\sigma_{X}=\sqrt{\frac{1}{k}\sum_{i=1}^{k}{\left(x_{i}-\bar{X}\right)}^{2}}$$

Pearson distance is defined as

(1) $$D_{P}\left(X,Y\right)=1-s_{X,Y}$$

The second distance is the Euclidean distance:

(2) $$D_{E}\left(X,Y\right)=\sqrt{\sum_{i=1}^{k}{\left(x_{i}-y_{i}\right)}^{2}}$$

The third distance measure is the squared Euclidean distance:

(3) $$D_{S\text{E}}\left(X,Y\right)=\sum_{i=1}^{k}{\left(x_{i}-y_{i}\right)}^{2}$$

### Gene order

As it is introduced before, a gene is associated with a vector that is derived from microarray data. In this way, a gene can be regarded as a virtual city whereby each coordinate is a vector. Two associated genes are more similar as the distance shortens between two virtual cities. As it is introduced at Section 1, an optimal (shortest) TSP route for a given set of virtual cities is the optimal gene order that is a permutation of all genes. In an optimal TSP route, closed cities are ordered together and the length of the route is that which is the shortest. In an optimal gene order, similar genes cluster together, and the quality of clustering is optimal globally. This is in contrast to many clustering methods that are only optimal locally.

Currently optimal gene order cannot be calculated perfectly because it is an NP-hard problem; only an approximation can be achieved. Therefore, we need a function to measure the quality of the approximation. The following function *Q*(*π*) is called a fitness function:

(4) $$Q\left(\pi \right)=\sum_{i=1}^{n}D\left(g_{\pi_{i}},g_{\pi_{i+1}}\right)$$

where *g_i _*denotes a vector associated with a gene, *π *denotes a gene order, *n *is the number of genes, *D*(*g_i_*, *g*_*i*+1_) is the distance between gene *g_i _*and gene *g*_*i*+1_, and $g_{\pi_{n+1}}=g_{\pi_{1}}$. The distance formula *D*(*g_i_*, *g*_*i*+1_) can be chosen from Pearson distance, Euclidean distance, squared Euclidean distance, Spearman distance, and other measurements.

Function *Q*(*π*) is a measurement of the quality of the gene order. The smaller the function value *Q*(*π*) is, the better the quality of the gene order *π *is.

However, the measurement of function *Q*(*π*) is not consistent with the fact of biology, and a true review of the quality of gene order depends on the review of a biologist. A biologist often reviews the quality of gene clustering by visually observing its heat map, and he or she often gets heuristic information from that heat map.

### Apply ACO to calculate optimal gene order

To generate the optimal gene order, ACO is applied as it is below:

**Step 1**: Use the distance formula to compute the distance between genes.

**Step 2**: Initialize the pheromone trails for all edges between genes (or virtual cities) and put *m *ants at different genes to travel. Pre-assign an iteration number *t*_max _and let *t *= 0, where *t *denotes the *t *- *th *iteration computation.

**Step 3**: *while*(*t *<*t*_max_)

{

**Step 3.1: **Each ant selects its next city according to the transition probability $p_{ij}^{k}\left(t\right)$.

The transition probability of the *k *- *th *ant from the *i *- *th *gene to *j *- *th *gene is defined as

$$p_{ij}^{k}\left(t\right)=\left\{\begin{array}{cc} \frac{\tau_{ij}^{\alpha }\left(t\right)\eta_{ij}^{\beta }\left(t\right)}{\underset{s\in allowed_{k}}{\sum }\tau_{is}^{\alpha }\left(t\right)\eta_{is}^{\beta }\left(t\right)}, & j\in allowed_{k}\\ 0 & otherwise \end{array}\right)$$

, where *allowed_k _*denotes the set of genes that can be accessed by the *k *- *th *ant; *τ_ij_*(*t*) is the pheromone value of the edge (*i, j*); *η_ij_*(*t*) is the local heuristic function and *η_ij_*(*t*) = 1/*d_ij_*, and where *d_ij _*are the distance between the *i *- *th *gene and *j *- *th *gene; the parameters α and β determine the relative influence of the trail strength and the heuristic information, respectively.

**Step 3.2**: After all ants finish their travels, all pheromone values *τ_ij_*(*t*) are updated according to the following formula.

$$\begin{array}{c} \tau_{ij}\left(t+1\right)=\left(1-\rho \right)\cdot \tau_{ij}\left(t\right)+\Delta \tau_{ij}\left(t\right)\\ \;\;\;\;\;\;\;\Delta \tau_{ij}\left(t\right)=\sum_{k=1}^{m}\Delta \tau_{ij}^{k}\left(t\right)\\ \;\;\;\;\;\;\;\;\;\Delta \tau_{ij}^{k}\left(t\right)=\left\{\begin{array}{c} \frac{Q}{L_{k}},e\left(i,j\right)\in L_{k}\\ 0,else \end{array}\right) \end{array}$$

, where *L_k _*is the length of the route passed by the *k *- *th *ant; *ρ *is the persistence of the trail; *Q *denotes constant quantity of pheromone; and *e*(*i, j*) represents the edge between gene *i *and gene *j*.

**Step 3.3**: *t *= *t *+ 1

}

**Step 4: **End procedure and select the TSP route that has the minimum length as the output.

### Apply GAs to calculate optimal gene order

As mentioned before, the calculation of gene order can be converted to TSP. To make GA fit to process TSP and gene order, the commonly used GA is modified a little. The modifications are listed below:

First, the roulette rule [46] is used to design selection probability.

Second, the crossover probability is set to be 1.0 in this paper. That is, the crossover will occur definitely.

Third, the mutation is designed to occur. Between the parent and mutated offspring, the one which has the better fitness value is selected as the genuine offspring, and the others are discarded.

The modified GA is described below:

**Step 1**: Initialization: Set the maximum iteration number to *t*_max_. The *t-th *iteration step is denoted by *t*. In this paper, the length of the chromosome is set to be the number of AD genes, which is denoted by *L*. The initial population is denoted by *P_old_*, and its size is set to be *N*.

**Step 2**: The next generation is denoted by *P_new_*, and it is initialized to be an empty set. In addition, a counter is used, which is denoted by *c*, and it is initialized to be 1.

**Step 3**: Selection

1. Calculate each chromosome's fitness value according to formula (4).

2. Calculate the proportion (ratio) of the fitness value of each chromosome.

3. A ratio is chosen by the roulette rule, and its associated chromosome is chosen too. According to this method, two chromosomes are chosen, which are denoted by *C*_1 _and *C*_2_.

**Step 4**: Crossover

1. Generate two random integer numbers between 1 and *L*, which are denoted by *C*_*po*int1 _and *C*_*po*int2_(*C*_*po*int1 _<*C*_*po*int2_), and where *C*_*po*int1 _and *C*_*po*int2 _are used to indicate the positions of two crossovers on chromosomes *C*_1 _and *C*_2_.

2. Denote the part of *C*_2 _from *C*_*po*int1 _to *C*_*po*int2 _as *C*_*t*2_, and copy it to the head of *C*_1_. The increased chromosome *C*_1 _is denoted by $C_{1}^{\prime }$.

Denote the part of *C*_1 _from *C*_*po*int1 _to *C*_*po*int2 _as *C*_*t*1_, and copy it to the head of *C*_2_. The increased chromosome *C*_2 _is denoted by $C_{2}^{\prime }$.

3. Find every gene that lies in chromosome *C*_*t*2 _and *C*_1_, which is denoted by *x *(i.e., *x *∊ *C*_*t*2 _∩ *C*_1_). Delete every *x *from *C*_1_, and add *C*_*t*2 _to the head of updated *C*_1 _(i.e., $C_{1}^{\prime }\leftarrow C_{t2}\cup \left(C_{1}-\left\{x\right\}\right)$). The updated $C_{1}^{\prime }$ is regarded as temporary offspring of *C*_1 _and denoted as *T*_*offspring*1_. Using the same method, the temporary offspring of *C*_2 _is generated, which is denoted as *T*_*offspring*2_.

**Step 5**: Mutation

Select a point on *T*_*offspring*1 _randomly as a mutation point, which is denoted by *M*_*po*int1_. Suppose the value of mutation point *M*_*po*int1 _is *V_old_*. Generate a random integer between 1 and *L *, which is denoted by *V_new_*. Set *V_new _*as the updated value of point *M*_*po*int1_.

Find the point at which value is equal to *V_new _*except point *M*_*po*int1_, and update its value as *V_old_*.

The chromosome *T*_*offspring*1 _is updated, and it is a true offspring.

Using the above method, chromosome *T*_*offspring*2 _can also be updated, and it is a true offspring.

**Step 6**: Add the two true offspring into the set *P_new_*, which represents the new population. Update the counter:

*c *= *c *+ 2, if *c *<*N*, go to Step 3, or else go to Step 7.

**Step 7**: Joint population *P_old _*and *P_new _*(i.e., P = *P_old _*∪ *P_new_*). Select *N *chromosomes from set *P *to cover the old population *P_old _*for which the fitness values are smaller than the other chromosomes.

**Step 8**: Increase the iteration step: *t *= *t *+ 1. If *t *<*t*_max_, and go to step 2, or else go to Step 9.

**Step 9**: End the algorithm and choose the chromosome that has the smallest fitness value from the last population *P_old _*as the output.

Kirk presented an improved GA (IGA) program [47], and it consists of three parts: mutation, group, and iteration.

#### Part I (operation of mutation)

Suppose there is a chromosome{*a*_1_, *a*_2_, *a*_3_, *a*_4_, *a*_5_, *a*_6_}, and it is a permutation of genes *a*_1_, *a*_2_, *a*_3_, *a*_4_, *a*_5 _and *a*_6_. Firstly, cut a sub-sequence from the chromosome randomly, and suppose it is {*a*_2_, *a*_3_, *a*_4_, *a*_5_}. Three types of mutations are listed below:

Flip operation *M_f_*:

Flip the gene positions of the sub-sequence. For example, $\left\{a_{2},a_{3},a_{4},a_{5}\right\}\overset{M_{f}}{\to }\left\{a_{5},a_{4},a_{3},a_{2}\right\}$.

Swap operation *M_s_*:

Swap the positions of the two terminal genes-$\left\{a_{2},a_{3},a_{4},a_{5}\right\}\overset{M_{s}}{\to }\left\{a_{5},a_{3},a_{4},a_{2}\right\}$.

Slide operation *M_l_*:

Shift the gene to the next position by a rotation-$\left\{a_{2},a_{3},a_{4},a_{5}\right\}\overset{M_{l}}{\to }\left\{a_{3},a_{4},a_{5},a_{2}\right\}$.

#### Part II (group)

Suppose *N *chromosomes, denoted by *s*_1_, *s*_2_, *s*_3_, ..., and *s_N_*, are generated randomly where *N *is divisible by 4. And all chromosomes are saved in a table *T *sequentially. In table *T*, every 4 chromosomes is grouped as a team sequentially. For every team, perform the following operations:

Firstly, select the chromosome with the minimal fitness value as seed, and discard the other three chromosomes.

Secondly, let the mutation operation *M_f_*, *M_s _*and *M_l _*act on the seed, respectively, and generate three mutated chromosomes.

Thirdly, all chromosomes in this team are updated as the seed and the three mutated chromosomes, which updates table *T*.

#### Part III (iteration computation)

An operation of a group is called an iteration computation. Within every iteration, an optimal chromosome will be generated for which the fitness value is minimal compared to the other *N *- 1 chromosomes. Suppose *R_t _*is the optimal chromosome of the *t-th *iteration. After all, iterations are performed on the set $\left\{R_{1},R_{2},...,R_{t_{\max}}\right\}$ for a given number of iteration *t*_max_. The solution is selected from $\left\{R_{1},R_{2},...,R_{t_{\max}}\right\}$, which has a minimal fitness value.

### Source data

In this paper, the AD microarray data was downloaded from GEO Datasets, NCBI [48], which includes 22283 genes. Four cases of control, incipient, moderate, and severe data are provided in the original data. Nine samples of control are organized to form a matrix with a size of 22283 lines by 9 columns. The format of this matrix is shown in Table 1. In this matrix, each line vector is a 9-dimensional vector that represents microarray data of a gene collected from nine different conditions. All line vectors form a data set.

**Table 1:** The illustration of organization of AD microarray data

AFFX -NAME	GSM 21215	GSM 2127	GSM 2128	GSM 21219	GSM 21220	GSM 21221	GSM 21226	GSM 21231	GSM 21232
BioB-5_at	8.937	9.941	8.986	9.305	9.366	8.781	9.236	9.35	9.386
BioB-M_at	9.278	10.56	9.55	10.08	10.23	9.355	9.915	10.27	10.37
BioB-3_at	7.92	9.033	8.71	8.993	9.353	8.381	8.716	9.481	9.299
BioC-5_at	10.18	11.46	10.49	10.76	10.88	10.25	10.52	10.87	10.91

*Each column of the data represents the result of one microarray test. Each line of the data represents the expression levels of the same gene under different conditions. All data was log-transformed.

Seven samples of incipient for each gene are selected to form a 7-dimensional vector, and the resulting 22283 vectors are used to form a data set; eight samples of moderate for each gene are selected to form an 8-dimensional vector and to form a data set; and seven samples of severe for each gene are selected to form a data set.

In addition, according to the usual practice, all data of the AD gene is log-transformed for smoothing.

### Computing parameters and environment

All data tested by GAs and ACO run on a personal computer, CPU (2): 2.99 GHZ, 3.0 GHZ; Memory: 1.0 GB.

The parameters of ACO are set below:

*α *= 1, *β *= 2, *ρ *= 0.7, *Q *= 100, *τ_ij_*(0) = 1, *m *= 50, *t*_max _= 100.

The parameters of GA are set below:

*t*_max _= 500, *M *= 400,

, where *t*_max _and *M *represents the maximal number of iterations and the size of populations, respectively.

The parameters for the improved genetic algorithm are set as below:

*t*_max _= 2000, *M *= 900.

In addition, in GA, parameter values of *t*_max _and *M *are smaller than parameters in IGA, respectively. The reason that the parameter value is different is that GA is much slower than IGA, and a high value of parameter will require excessive GA program running time.

## Results and discussion

**Figure 1:**
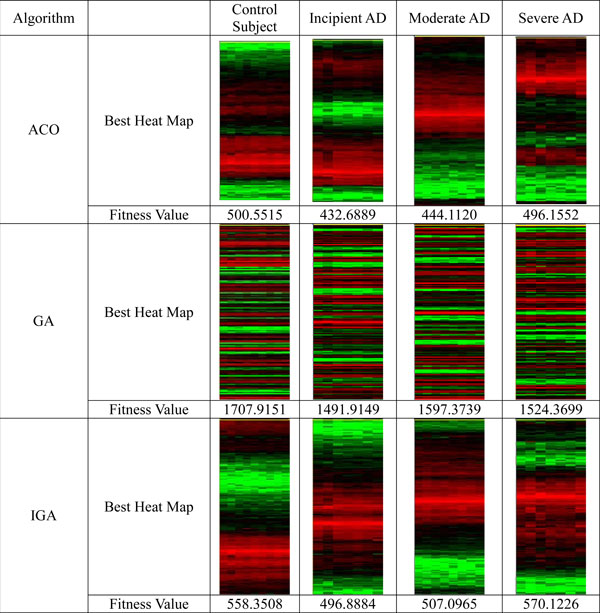
**The comparison of the quality of gene order generated by ACO and GA using Euclidean distance**. *Ancillary information for figures:1. All microarray data are downloaded from [48], and the data from the 1^st ^line to 300^th ^line are used to do experiment and for other figures and tables. 2. Every heat map is the optimal gene order, which has the smallest value of fitness function and was selected from tests performed over 40 times. In addition, the distance formula used in the fitness function (see formula 4) is the Euclidean Distance. 3. All of the figures listed in this paper are generated by TreeView, which was developed by Dr Eison, and is downloaded from the website: http://rana.lbl.gov/downloads/TreeView/TreeView_vers_1_60.exe. 4. Because most of the expression levels of the AD gene data are larger than zero, the average value of every column is subtracted when the heat map is shown. Otherwise, all heat maps are red, and the display is incorrect.

The results are showed in Figure 1 to Figure 3, and Table 2 to Table 3. From these figures and tables, we discovered that:

**Figure 2:**
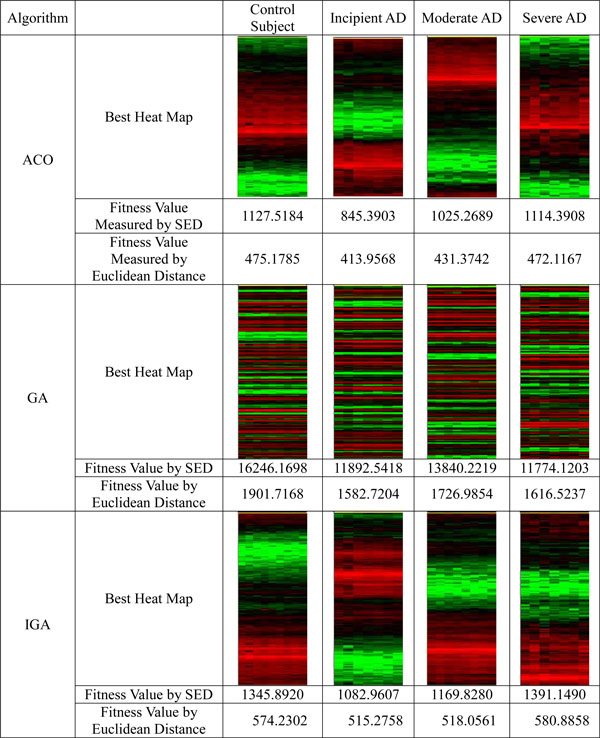
**The comparison of the quality of gene order generated by ACO and GA using squared Euclidean distance formula**.

**Figure 3:**
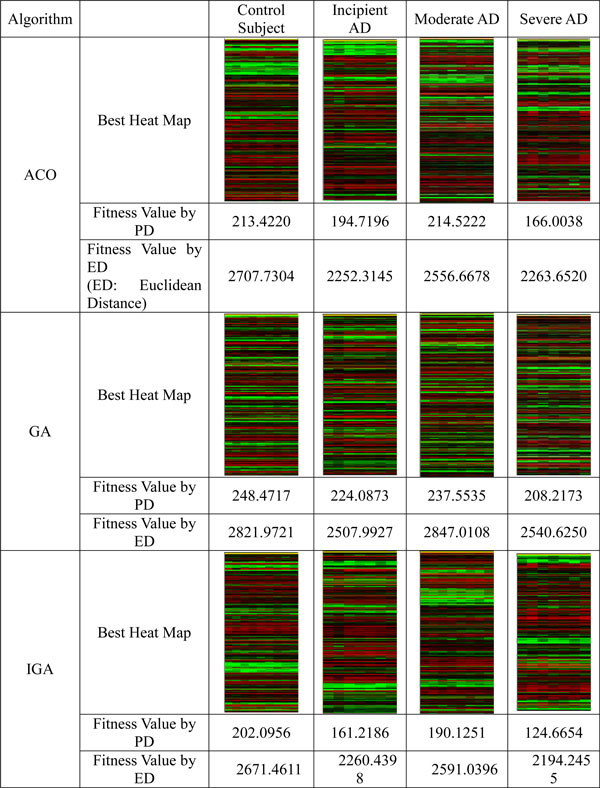
**The comparison of the quality of gene order generated by ACO and GA using Pearson distance formula**.

**Table 2:** The statistical comparison of the quality of gene order

Algorithm	Distance	Control man	Incipient patient	Moderate patient	Severe patient
ACO	ED	507.9163	442.7255	459.7381	504.0716
GA	ED	1800.9287	1582.5394	1689.2580	1604.3304
IGA	ED	566.0912	508.6311	516.0917	579.3226

ACO	SED	484.8221	419.8804	437.9346	479.5701
GA	SED	1916.9891	1679.9281	1789.6030	1682.0008
IGA	SED	576.9810	521.2992	529.8852	593.4252

ACO	PD	2737.5938	2233.1848	2518.7568	2167.4011
GA	PD	2882.9409	2532.2205	2708.5082	2515.8520
IGA	PD	2712.5501	2319.1112	2513.9173	2218.1910

**Notation: **ED: Euclidean Distance; PD: Pearson Distance; SED: Squared Euclidean Distance

**Ancillary information: **all data in this table is the value of the fitness function, and it is the average of 40 times of tests. In addition, the distance formula used to calculate fitness value is ED. Every data in Table 5 corresponds to an average runtime.

**Table 3:** The statistical comparison of the runtime of ACO, GA and IGA

Algorithm	Distance	Control man	Incipient patient	Moderate patient	Severe patient
ACO	ED	122.0545	121.8582	121.8611	121.8653
GA	ED	580.8345	586.7355	588.9012	586.7427
IGA	ED	133.0079	131.2152	140.4218	139.1710

ACO	SED	109.8382	110.0110	109.7321	110.2532
GA	SED	186.4143	184.5551	185.1629	185.7899
IGA	SED	126.8957	126.9276	126.9757	127.0232

ACO	PD	123.0438	122.8454	122.6719	122.6450
GA	PD	186.9550	187.5644	187.0732	188.4089
IGA	PD	129.8745	127.7448	127.0051	126.4476

**Notation: **ED: Euclidean Distance; PD: Pearson Distance; SED: Squared Euclidean Distance

**Ancillary Information: **Every runtime in this table is the average of 40 times of tests. In addition, every runtime corresponds to a fitness value i listed at Figure 3.

(1) ACO was better suited than GA to calculate the gene order of the AD genes tested in this paper.

(2) Both for ACO and GAs, the use of different distance formulas generated a different quality of gene order. The squared Euclidean distance generated the best quality overall compared with the Pearson distance and Euclidean distance.

Pearson distance is a popular distance formula that is commonly used to calculate gene order. However, we found that Pearson distance is not the optimal distance formula for the calculation of gene order associated with AD genes. In this paper, the original data is not normalized, the reason for which is explained below:

Suppose two genes and their associated vectors are *X *= (*x*_1_, *x*_2_, ..., *x*_*k*_) and *Y *= (*y*_1_, *y*_2_, ..., *y*_*k*_). If all components of the vector are normalized, they become small real value that is less than 1.0. Value $S=\sum_{i=1}^{k}{\left(x_{i}-y_{i}\right)}^{2}$ is small, and it is close to zero if the two genes are very similar. Then the value of the square-root $\sqrt{S\;}$ has a big error because it must be expressed as base operations (+, -, ×, and ÷) to approximate. That is why Pearson distance, Euclidean distance and other distance formulas generate lower qualities of gene order calculation compared with squared Euclidean distance.

## Conclusion

With AD being the most common form of senile dementia, the study of AD-associated genes is an imperative research subject. One important branch of an AD gene study is to cluster AD genes with the highest quality; gene order generates a better quality of clustering than other methods in general. In addition, our results of the experiment support the following conclusion: ACO is better than GA in AD gene order computation. Further, the following computational features were revealed in our study: For both ACO and GA, different distance formulas generated a different quality of gene order. Compared to Pearson distance and Euclidean distance, the squared Euclidean distance generated the best quality of AD gene order. Although Pearson distance commonly used tool, it is less optimal in AD gene order computation when employed in both ACO and GA methods.

## Competing interests

The authors declare that they have no competing interests.

## Authors' contributions

BH formulated the computing framework in this paper with CP together. GJ performed computing and drafted this manuscript with CP together. SW wrote part of background section for this manuscript. QL, ZC, CRV, JTR, and YD assisted the study and provided some suggestions. XH initiated the project, provided the guidance for the study, and performed the final editing for the manuscript. All authors have read and approved the final manuscript.
